# A case report of a multivessel coronary vasospasm that responded to IV diazepam

**DOI:** 10.1097/MD.0000000000044267

**Published:** 2025-09-12

**Authors:** Fares Abboud, Mustafa Zain, Ahmad Alsaadi, Farah Haneyah, Amal Jama, Ghassan Bayat

**Affiliations:** a Faculty of Medicine, Damascus University, Damascus, Syrian Arab Republic; b Department of Cardiology, Faculty of Medicine, Al Mouwasat University Hospitals, Damascus University, Damascus, Syrian Arab Republic; c Al-Balqa Applied University, Salt, Jordan; d Gazi University Faculty of Medicine, Ankara, Turkey; e Plymouth University Hospitals NHS Trust, Plymouth, UK.

**Keywords:** anxiety-induced vasospasm, coronary artery vasospasm, intravenous diazepam, multivessel vasospasm, psychological stress

## Abstract

**Rationale:**

Coronary artery vasospasm (CAVS) is a significant cause of acute chest syndrome, often mimicking coronary artery disease on angiography, which may lead to unnecessary interventions like percutaneous coronary intervention or coronary artery bypass grafting. This case report aims to highlight the potential therapeutic role of intravenous (IV) diazepam in managing multivessel CAVS unresponsive to standard vasodilators, emphasizing the influence of psychological stress in such cases.

**Patient concerns:**

A 32-year-old male, a heavy smoker with a history of anxiety disorder and no familial coronary artery disease history, presented with angina.

**Diagnoses:**

Initial evaluations, including electrocardiogram, echocardiogram, and laboratory tests, were normal, but an exercise stress electrocardiogram was positive. Coronary angiography revealed severe lesions in the proximal left anterior descending and right coronary artery diagnosing multivessel CAVS.

**Interventions:**

The patient was unresponsive to intracoronary nitroglycerin and verapamil. During a panic attack in the procedure, 5 mg IV diazepam was administered.

**Outcomes:**

Treatment lead to symptom resolution and normal coronary arteries on repeat angiography, without plaques or stenosis.

**Lessons:**

This case demonstrates multivessel CAVS refractory to standard vasodilators but responsive to IV diazepam, as evidenced by angiographic resolution. The temporal association with a panic attack suggests a link to psychological stress, aligning with literature on stress-related cardiovascular events. Limitations include the single-case design, lack of provocation testing, and inability to definitively establish causality between anxiety and vasospasm, necessitating larger studies to confirm IV diazepam’s efficacy. Multivessel CAVS may respond to IV diazepam when standard treatments fail, particularly in cases associated with psychological stress. This case underscores the need for further research to validate the therapeutic potential of IV benzodiazepines in managing CAVS and to explore the interplay between psychological stress and cardiovascular events.

## 1. Introduction

Coronary artery vasospasm (CAVS) is a critical cause of acute chest syndrome, resulting from transient coronary artery constriction that impairs blood flow.^[1]^ It is diagnosed in approximately 54.7% of patients presenting with chest pain and no significant coronary artery disease (CAD) when assessed with acetylcholine (ACH) provocation testing.^[2]^ Multivessel CAVS, characterized by simultaneous spasms in multiple coronary arteries, is less prevalent but poses significant diagnostic challenges, as it can mimic diffuse CAD on coronary angiography, potentially leading to unnecessary interventions such as percutaneous coronary intervention or coronary artery bypass grafting.^[3]^ Diagnosis typically relies on coronary angiography, often supplemented by ACH provocation testing to confirm vasospasm, though this carries procedural risks and is not universally performed.^[1]^ Standard treatment involves intracoronary nitroglycerin to relieve acute vasospasm and calcium channel blockers, such as verapamil, for long-term prevention, but some cases remain refractory to these therapies.^[1]^ Emerging evidence suggests that psychological stress, including severe anxiety, may contribute to CAVS, potentially through mechanisms like microvascular dysfunction or catecholamine surges.^[4]^ Despite this, the role of benzodiazepines, such as intravenous (IV) diazepam, in managing CAVS remains underexplored. This case report presents a novel instance of multivessel CAVS unresponsive to standard vasodilators but effectively resolved with IV diazepam, highlighting a potential therapeutic role for benzodiazepines in specific clinical scenarios driven by psychological stress.

## 2. Case presentation

A 32-year-old male, a heavy smoker with a history of anxiety disorder and no family history of CAD, presented with pressure-like chest pain on minimal exertion, shortness of breath, and dyspnea (grade 3 on the modified Medical Research Council scale). Clinical examination showed a blood pressure of 135/75 mm Hg, oxygen saturation of 98%, respiratory rate of 18 breaths per minute, and heart rate of 80 beats per minute. The patient’s body mass index was 30 kg/m^2^. Physical examination was otherwise unremarkable.

**Figure 1.**
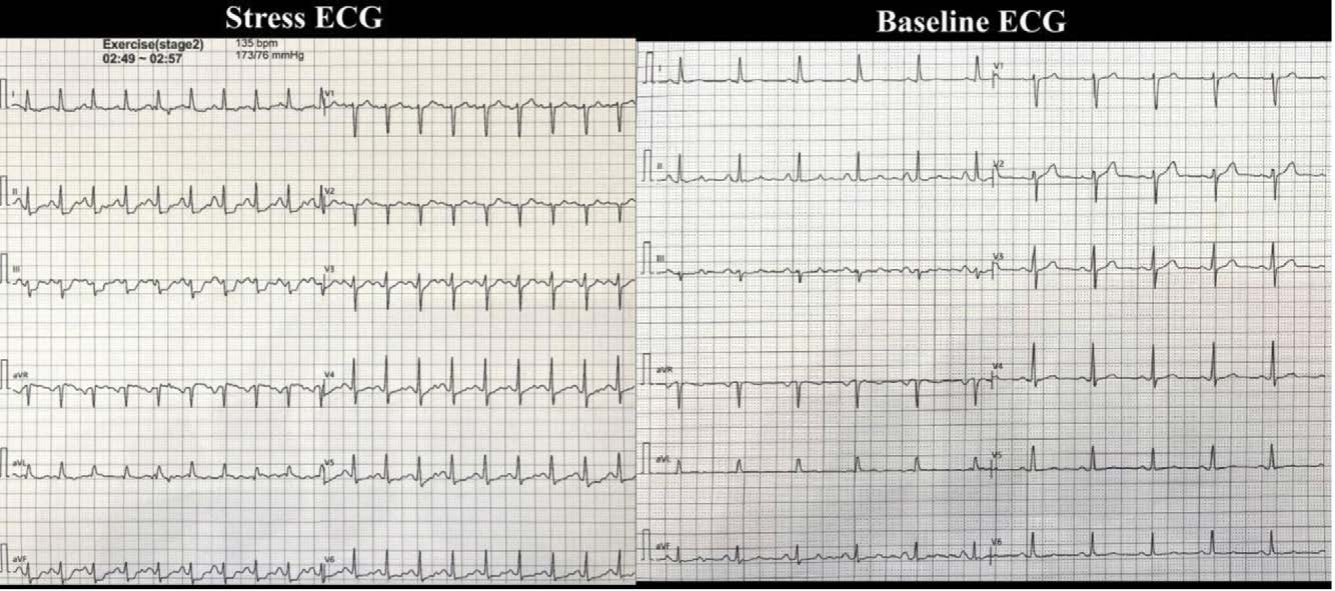
On the right is the baseline ECG showing normal patterns. On the left is the stress ECG showing ST depression in leads (II, III, AVF, V4, V5, and V6). ECG = electrocardiogram.

**Figure 2.**
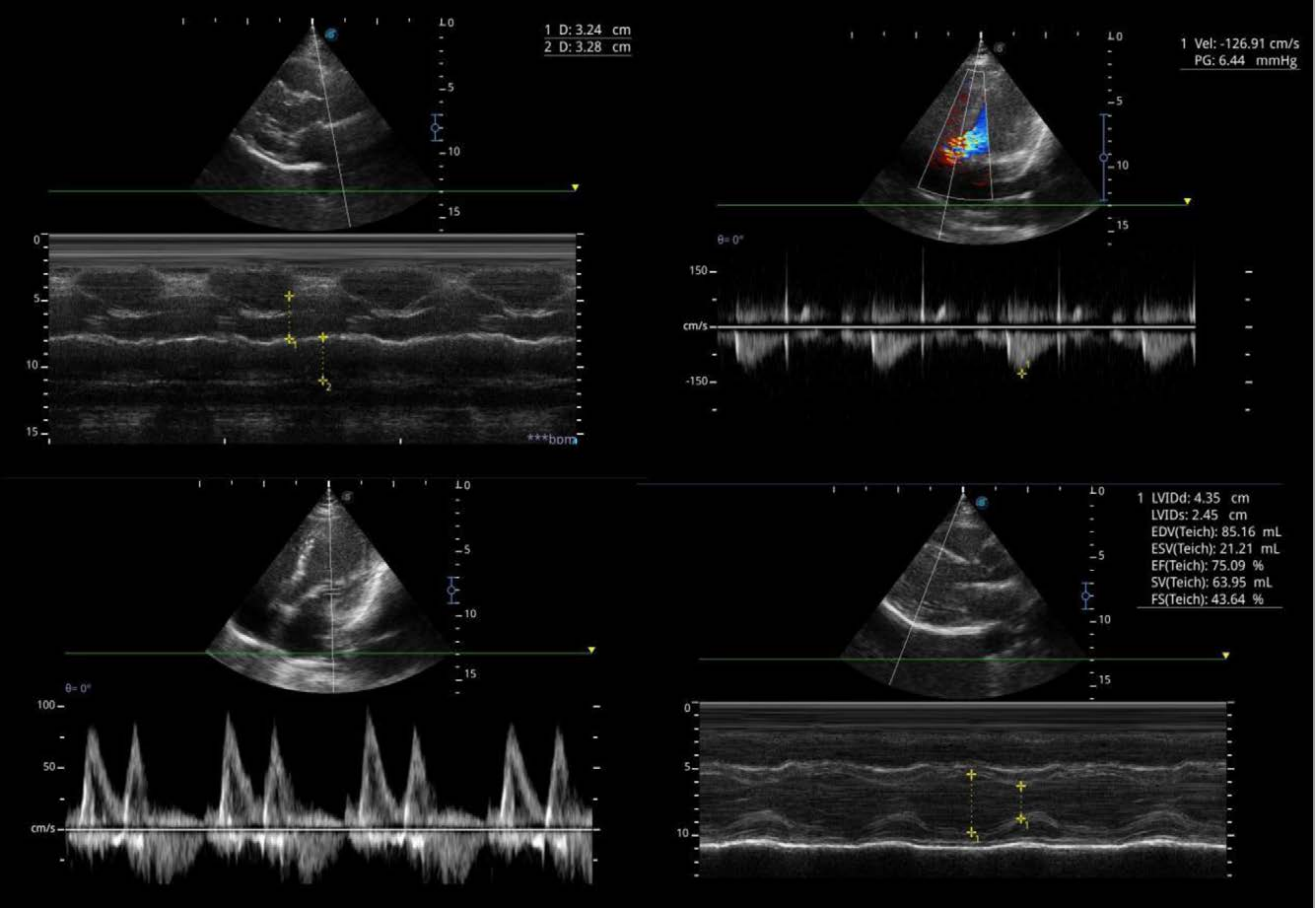
Baseline echocardiogram showing no structural or functional abnormalities.

**Figure 3.**
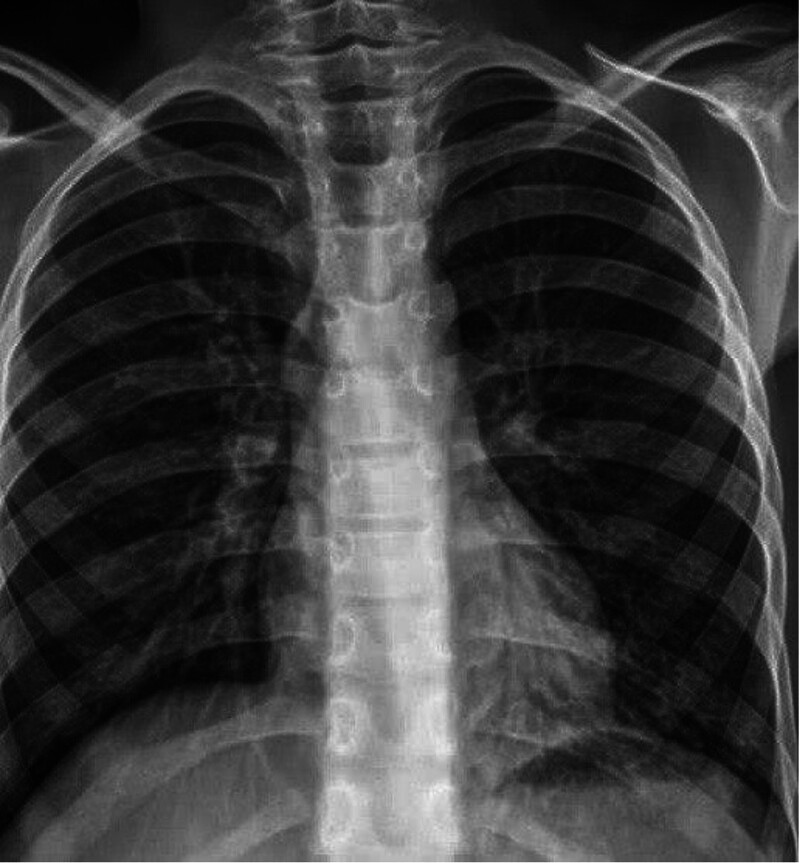
Simple chest X-ray showing no abnormalities.

Initial diagnostic tests included an electrocardiogram (ECG) in Figure 1 and echocardiogram in Figure 2, both normal, with the echocardiogram showing no structural or functional abnormalities. A chest X-ray showed clear lungs with no visible nodules, tumors, or masses provided in Figure 3.

Laboratory results are shown in Table 1:

**Table 1:** Respective serum levels on time of admission.

Serum blood levels			
WBC: 4.3 10^9^/L	Hemoglobin: 13.9 g/dL	MCV: 82 fl	MCH: 31 pg/cell
PLT: 295 10^9^/L	Creatinine: 0.7 mg/dL	BUN: 4 mmol/L	Glucose (fasting): 92 mg/dL
Na: 139 mEq/L	K: 3.9 mmol/L	INR: 1	PTT: 31 s
ALT: 22 U/L	AST: 25 U/L	Troponin I: 0.01 ng/mL (negative)	Cholesterol (total): 179 mg/dL
TG: 160 mg/dL	LDL: 95 mg/dL	HDL: 52 mg/dL	
ALT = alanine aminotransferase, AST = aspartate aminotransferase, BUN = blood urea nitrogen, HDL = high-density lipoprotein, INR = international normalized ratio, K = potassium, LDL = low-density lipoprotein, MCH = mean corpuscular hemoglobin, MCV = mean corpuscular volume, Na = sodium, PLT = platelet count, PTT = partial thromboplastin time, TG = triglycerides, WBC = white blood cell count.			

An exercise stress ECG was positive, with the patient developing severe chest pain and 2.5 mm horizontal ST-segment depression in the inferior leads after 3 minutes, suggesting ischemia as shown in Figure 1. No testing for ventricular fibrillation was performed, as the clinical presentation and ECG findings did not indicate an arrhythmic event.

The patient’s symptoms raised suspicion for CAD, Prinzmetal’s angina, or microvascular dysfunction. The absence of troponin elevation and normal echocardiogram made acute myocardial infarction unlikely. The positive stress ECG and heavy smoking history supported possible CAD, but the lack of family history and normal lipid profile reduced this likelihood. Prinzmetal’s angina was considered due to the transient nature of symptoms, but the multivessel involvement and response to diazepam favored anxiety-associated CAVS.

**Figure 4.**
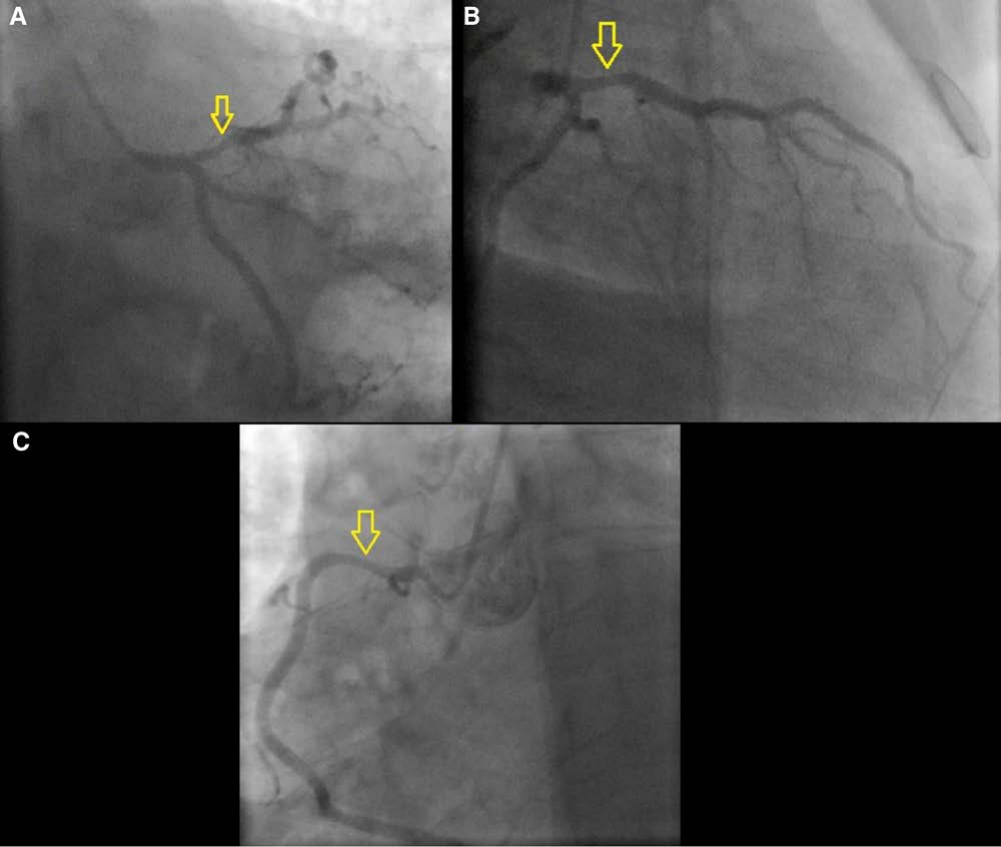
(A, B) Coronary angiography revealing severe proximal left anterior descending (LAD) stenosis (arrow) before IV diazepam administration, indicative of multivessel vasospasm in a 32-year-old male with anxiety and angina, unresponsive to intracoronary nitroglycerin (100 mcg × 2) and verapamil (0.5 mg). (C) Coronary angiography demonstrating significant proximal right coronary artery (RCA) stenosis (arrow) prior to IV diazepam, highlighting the extent of vasospasm in the same patient, with no improvement following standard vasodilator therapy. IV = intravenous.

**Figure 5.**
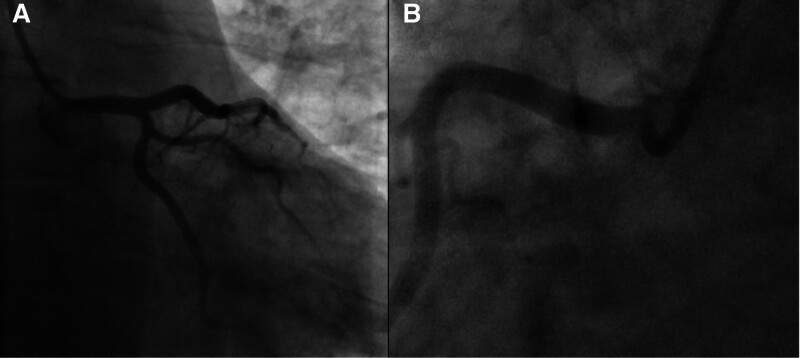
(A) Post-IV diazepam (5 mg) coronary angiography showing normal left coronary arteries, with the LAD now free of stenosis, illustrating the resolution of vasospasm following anxiety management. (B) Post-IV diazepam coronary angiography depicting a normalized proximal RCA, confirming the reversal of vasospasm and absence of atherosclerotic plaques, supporting the anxiety-induced etiology. LAD = left anterior descending, RCA = right coronary artery, IV = intravenous.

Coronary angiography revealed severe stenosis in the proximal left anterior descending (LAD) and right coronary artery (RCA) (Fig. 4), suggestive of multivessel vasospasm. Intracoronary nitroglycerin (200 mcg total, 100 mcg per artery) and verapamil (0.5 mg per artery) were administered without effect. During the procedure, the patient experienced a panic attack, treated with 5 mg IV diazepam. Symptoms resolved, and repeat angiography showed normal coronary arteries without plaques or significant stenosis (Fig. 5). No cardiac ultrasound (beyond the initial echocardiogram), PET, or MRI was performed, as the clinical presentation and angiographic findings were sufficient for diagnosis.

**Figure 6.**
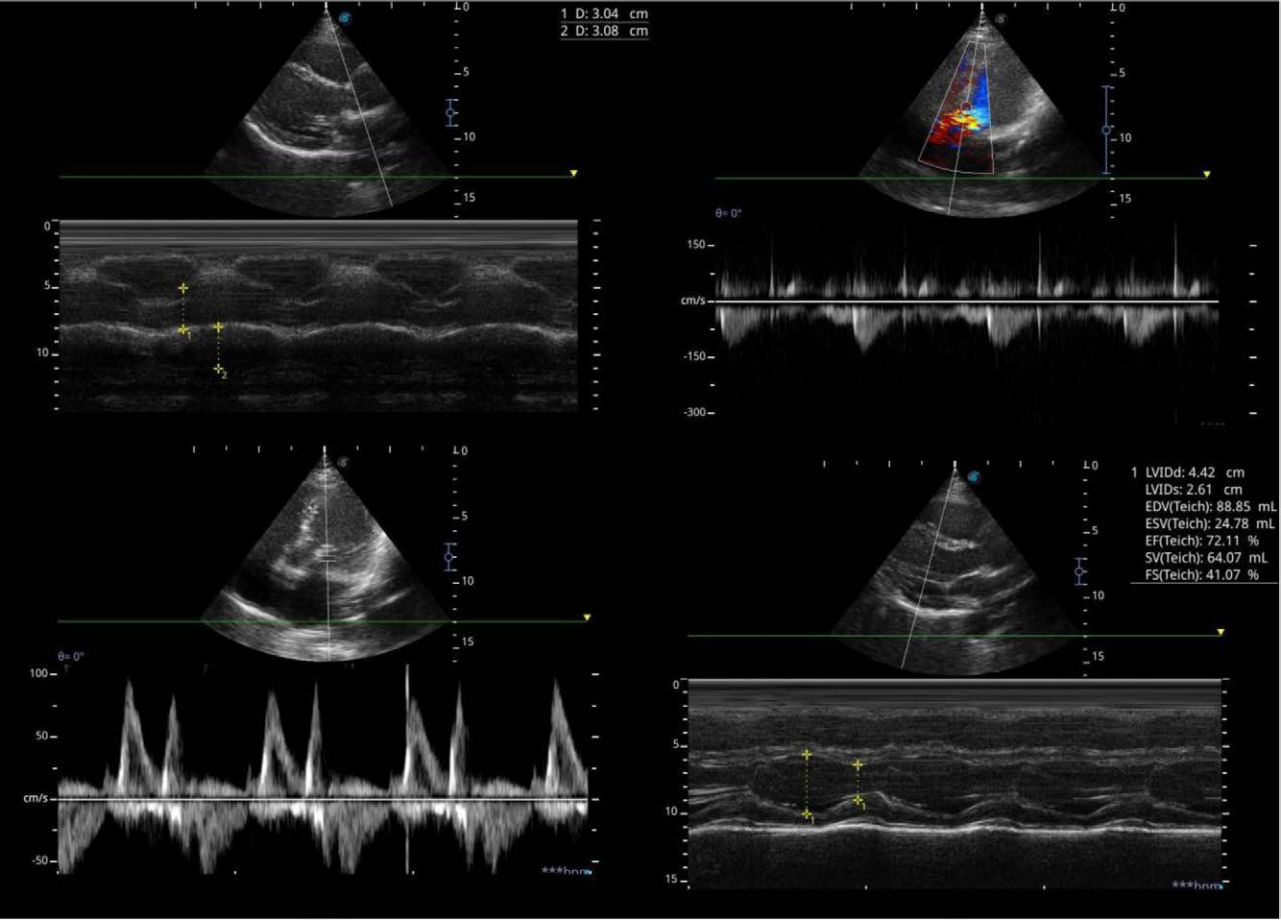
Regular follow-up echocardiogram showing no adverse effects on the cardiac function.

The patient was discharged on isosorbide mononitrate (20 mg daily) and nebivolol (5 mg daily) to prevent recurrent vasospasm and referred for psychological counseling to address anxiety. Follow-up at 3 months revealed no recurrence of chest pain, with the patient reporting improved anxiety symptoms but with nonadherence to cognitive behavioral therapy. Follow-up echocardiogram showing no functional abnormalities (Fig. 6). Informed consent was taken from the patient for the publication of this case report.

## 3. Discussion

This case report describes a 32-year-old male with multivessel CAVS that was refractory to standard vasodilators, including intracoronary nitroglycerin and verapamil, but resolved dramatically following the administration of 5 mg IV diazepam during a panic attack. The primary outcome was the complete resolution of angiographic lesions in the proximal LAD and RCA post-diazepam administration, as evidenced by repeat coronary angiography (Figs. 4 and 5). Secondary outcomes included symptom relief during the procedure, no recurrence of chest pain at 3-month follow-up, and a normal follow-up echocardiogram (Fig. 6), indicating preserved cardiac function. The temporal association between the panic attack and the resolution of angiographic lesions (Figs. 4 and 5) suggests that psychological stress may have contributed to the vasospasm, though a definitive causal link cannot be established in this single-case study. This observation aligns with existing literature linking psychological stress to cardiovascular events, including myocardial ischemia and vasospasm.^[4,5]^ The case highlights the potential utility of IV benzodiazepines in managing select cases of CAVS, particularly when standard therapies fail, but it also underscores the need for larger studies to validate this approach.

CAVS is a well-documented cause of acute chest syndrome, characterized by transient coronary artery constriction that can mimic obstructive CAD on angiography.^[1]^ Multivessel CAVS, as observed in this patient with severe stenoses in the proximal LAD and RCA, is less common but can lead to significant diagnostic and therapeutic challenges.^[3]^ The patient’s heavy smoking history is a recognized risk factor for CAVS, as smoking is associated with endothelial dysfunction and heightened vascular reactivity.^[6]^ Other predictors of CAVS, such as elevated high-sensitivity C-reactive protein or low diastolic blood pressure, were not assessed in this case, limiting a comprehensive risk profile.^[6]^ The absence of familial CAD history and a relatively normal lipid profile (low-density lipoprotein: 95 mg/dL, high-density lipoprotein: 52 mg/dL) reduced the likelihood of atherosclerotic disease, further supporting vasospasm as the primary etiology.

The failure of intracoronary nitroglycerin (200 mcg total) and verapamil (0.5 mg per artery) to relieve the vasospasm is notable, as nitrates and calcium channel blockers are the cornerstone of CAVS treatment.^[1]^ This refractoriness may reflect a distinct pathophysiological mechanism, possibly driven by the patient’s acute psychological stress during the procedure. The administration of IV diazepam, prompted by a panic attack, resulted in rapid symptom resolution and normalization of coronary arteries on repeat angiography, suggesting that addressing the patient’s acute anxiety-induced stress was critical. This finding echoes a prior report by Vidovich et al, where a 61-year-old woman with anxiety and depression experienced angina relief with diazepam and sertraline, implicating psychological factors in vasospasm.^[7]^ The mechanism remains speculative, but catecholamine surges during panic attacks may induce vasospasm, potentially via microvascular dysfunction or heightened sympathetic activity.^[4]^ Diazepam’s anxiolytic properties likely mitigated this cascade, restoring vascular tone.

The decision not to perform ACH provocation testing, which could have confirmed the vasospasm’s etiology, was based on the clear angiographic resolution and clinical improvement post-diazepam, avoiding additional procedural risks.^[1]^ Similarly, advanced imaging modalities such as cardiac MRI or PET were not pursued, as the initial echocardiogram was normal (Fig. 2), and the absence of troponin elevation ruled out myocardial damage. The lack of testing for ventricular fibrillation was justified, as the patient exhibited no arrhythmic symptoms, and the stress ECG findings (2.5 mm Hg horizontal ST-segment depression) were consistent with ischemia rather than an arrhythmia^[2]^ (Fig. 1). The differential diagnosis included Prinzmetal’s angina, CAD, and microvascular dysfunction, but the multivessel involvement, lack of nocturnal symptoms, and response to diazepam favored stress-associated CAVS over other etiologies.^[1]^

This case raises several critical considerations for clinical practice. First, it underscores the importance of recognizing psychological stressors in patients with atypical chest pain, particularly younger individuals with risk factors like smoking but no significant CAD history. The patient’s history of an anxiety disorder and the onset of a panic attack during angiography were pivotal in guiding management. Second, it highlights the limitations of standard vasodilator therapy in certain cases of CAVS and suggests that benzodiazepines may serve as an adjunctive or rescue therapy in stress-driven scenarios. However, several limitations must be acknowledged. The single-case design restricts generalizability, and the absence of provocation testing limits definitive confirmation of vasospasm etiology. Additionally, the lack of comprehensive risk factor assessment (e.g., high-sensitivity C-reactive protein) and the inability to establish causality between anxiety and vasospasm represent constraints. The patient’s nonadherence to cognitive behavioral therapy during follow-up further complicates long-term management assessment. Large cohort studies are essential to establish the prevalence of stress-associated CAVS and the efficacy of IV diazepam, addressing these limitations.

The patient’s follow-up at 3 months, with no recurrence of chest pain and improved anxiety symptoms, with a normal echocardiogram (Fig. 6), supports the role of appropriate management. The absence of plaques or stenosis on repeat angiography (Fig. 5) further confirms that the initial lesions were vasospastic rather than atherosclerotic, reinforcing the diagnosis of CAVS.^[3]^ However, the rarity of multivessel CAVS and the unique response to diazepam limit the generalizability of this case, necessitating further research to delineate the role of benzodiazepines in CAVS management.

In summary, this case illustrates a rare presentation of multivessel CAVS unresponsive to standard therapies but responsive to IV diazepam, with a possible association with psychological stress. While the findings are compelling, they are hypothesis-generating, and controlled studies are needed to confirm the therapeutic potential of benzodiazepines in similar scenarios.

## 4. Conclusion

This case report illustrates that multivessel CAVS, refractory to standard vasodilators such as intracoronary nitroglycerin and verapamil, may respond effectively to IV diazepam, particularly when associated with acute psychological stress, as observed during a panic attack. The rapid resolution of angiographic lesions following diazepam administration suggests a potential role for benzodiazepines as an adjunctive therapy in select cases of CAVS where conventional treatments fail. However, the single-case nature of this report, lack of provocation testing, and incomplete risk factor assessment limit its generalizability, and large-scale, controlled studies are essential to confirm the efficacy, safety, and optimal indications for IV diazepam in managing stress-associated CAVS. These findings emphasize the importance of considering psychological factors in the evaluation and treatment of atypical chest pain and highlight the need for integrated approaches combining pharmacological and psychological interventions to optimize patient outcomes.

## Author contributions

**Conceptualization:** Fares Abboud, Mustafa Zain, Ahmad Alsaadi.

**Formal analysis:** Fares Abboud, Mustafa Zain, Ahmad Alsaadi.

**Investigation:** Fares Abboud, Mustafa Zain, Ahmad Alsaadi.

**Methodology:** Mustafa Zain.

**Validation:** Mustafa Zain, Ahmad Alsaadi.

**Visualization:** Mustafa Zain, Ahmad Alsaadi.

**Writing – original draft:** Fares Abboud, Mustafa Zain, Ahmad Alsaadi, Farah Haneyah, Amal Jama, Ghassan Bayat.

**Writing – review & editing:** Fares Abboud, Mustafa Zain, Ahmad Alsaadi, Farah Haneyah, Amal Jama, Ghassan Bayat.
